# Influence of body mass index on postoperative complications after thymectomy in myasthenia gravis patients

**DOI:** 10.18632/oncotarget.19189

**Published:** 2017-07-12

**Authors:** Xu-Dong Liu, Ming-Rui Shao, Lei Sun, Lin Zhang, Xin-Shan Jia, Wen-Ya Li

**Affiliations:** ^1^ Department of Thoracic Surgery, The First Affiliated Hospital of China Medical University, Shenyang, Liaoning Province, China; ^2^ Department of Rheumatology and Immunology, The First Affiliated Hospital of China Medical University, Shenyang, Liaoning Province, China; ^3^ Department of Pathology, The First Affiliated Hospital of China Medical University, Shenyang, Liaoning Province, China; ^4^ Department of Pathology, College of Basic Medical Sciences, China Medical University, Shenyang, Liaoning Province, China

**Keywords:** myasthenia gravis, body mass index, postoperative complications, thymectomy

## Abstract

**Objectives:**

It is not clear whether being overweight or obese influences postoperative complications in myasthenia gravis (MG) patients. We retrospectively investigated an association between body mass index (BMI) and postoperative complications in MG.

**Materials and Methods:**

Fifty-nine MG patients who had undergone transsternal thymectomy were classified as low or high BMI based on the criteria for Asian-Pacific populations. An association between BMI and complications was analyzed.

**Results:**

MG patients with high BMI had significantly higher rates of major adverse complications (*P* = 0.033), postoperative respiratory failure (*P* = 0.045), and longer postoperative hospitalization (*P* = 0.005). The optimal cutoff value of BMI for postoperative respiratory failure was 23.3 kg/m^2^, with a sensitivity of 75.0% and a specificity of 64.7% (*P* = 0.046).

**Conclusions:**

MG patients with a BMI indicating overweight or obesity have a higher risk of postoperative complications after thymectomy. Thus, close monitoring must be performed when surgery is necessary.

## INTRODUCTION

Worldwide, it is estimated that 110 million children and adolescents in 2013, and 640 million adults in 2014, were overweight or obese. In 2013 about 4.5 million deaths were related to being overweight or obese [1]. Mounting evidence indicates that obesity is linked to a high risk of multiple serious conditions, including type 2 diabetes, cardiovascular disease, metabolic disturbances, and several cancers [2–7]. Considering the potential for obesity to become a global health crisis, it is not only a chronic health condition but also a disease [8].

Myasthenia gravis (MG) is a common autoimmune disorder that results from neuromuscular transmission abnormalities via several types of autoantibodies. MG is clinically characterized by muscle weakness and fatigue upon mild exertion [9–11]. The annual incidence of MG is 8–10 persons per million, and the prevalence rate is 150–250 per million [12]. The weakness may be localized or generalized, and the eye muscles are the most affected. MG can become life threatening when respiratory insufficiency occurs, due to involvement of the intercostal and diaphragmatic muscles.

Thymectomy has been shown to benefit MG patients with thymoma or thymic hyperplasia, and also in some patients with non-thymomatous MG [13–15]. However, it is noted that special attention must be paid to MG patients undergoing surgery, since administration of muscle relaxants may aggravate muscle weakness [14].

It has been reported that obesity is associated with higher rates of postoperative complications, but this remains controversial [16–18]. Hypoxia can be locally and systemically induced by excessive adipose tissue, and MG is vulnerable to oxygen. We therefore investigated whether body mass index (BMI), an accepted measure of obesity, is associated with postoperative problems in patients with MG, and especially severe complications such as postoperative respiratory failure (PRF).

## RESULTS

### Patient characteristics

The study population comprised 59 patients (25 male, 34 female), with a mean age of 48 years (range 15–70 years; Table 1). The disease course varied from 5 days to 10 years (mean, 234 days). Of these 59 patients, 24 had a mass ≥ 4.6 cm (mean size). Anti-acetylcholine receptor antibody titers ranged from 1.1 to 12.5 nM, evaluated by radioimmunoassay.

**Table 1:** Basic clinical characteristics of patients with MG stratified by low and high BMI

variables	Number	BMI ( kg/m^2^)		χ^2^	*P*-value
		low group (< 23.0)	High group (≧ 23.0)		
**Age (y)**				0.272	0.602
< 48	26	14	12		
≧ 48	33	20	13		
**Gender**				0.100	0.752
Male	25	15	10		
Female	34	19	15		
**Smoking History**				2.508	0.113
Yes	13	5	8		
No	46	29	17		
**Course (d)**				0.002	0.967
< 234	45	26	19		
≧ 234	14	8	6		
**Tumor size (cm)**				0.393	0.531
< 4.6	35	19	16		
≧ 4.6	24	15	9		
**WHO histology^a^**				2.170	0.825
Hyperplasia	11	5	6		
Type A	5	4	1		
Type AB	7	4	3		
Type B1	9	6	3		
Thymoma B2	17	9	8		
Thymoma B3	10	6	4		
**Masaoka stage**				0.003	0.956
I + II stage	47 (23 + 24)	27 (13 + 14)	20 (10 + 10)		
III + IV stage	12 (11 + 1)	7 (7 + 0)	5 (4 + 1)		

^a^thymus hyperplasia, type A1, AB, B1, B2, B3 thymoma.

The WHO histology classifications were as follows: thymus hyperplasia in 11 cases; type A thymoma in 5 cases; type AB in 7 cases; type B1 in 9 cases; type B2 in 17 cases; and type B3 in 10 cases; Regarding Masaoka stages: 23, 24, 11, and 1 patient were at stages I, II, III, and IV, respectively.

The low- and high-BMI groups were statistically comparable with regard to each clinical characteristic (Table 1).

### Association between BMI and postoperative complications

Of the 59 patients, 9 (15.3%) experienced severe postoperative complications that required rescue treatment or secondary surgery: 8 with respiratory failure due to various causes, and 1 with sternal dehiscence (Table 2). Using a logistic regression model, we investigated associations between each of the clinical parameters and major adverse complications, and found that only BMI was a statistical factor (Table 3).

**Table 2:** Detailed clinical records of the nine MG patients with severe complications

Case	sex	Age (y)	course	BMI	smoke	Tumor (cm)	Histology	Masaoka Stage^a^	Complications	Incubation^b^ (d)	Outcome^c^
1	M	50	1 year	27.0	yes	2.4	B2	II	MC	2	Survived
2	F	69	3 months	23.3	No	8.0	A	I	MC; PI; MODS	18	Died
3	F	52	4 months	26.2	Yes	5.5	B1	I	MC	7	Survived
4	M	44	3 months	25.8	No	3.0	AB	I	Sternal dehiscence	0	Survived ^d^
5	F	62	1 year	30.1	No	2.0	B1	II	MC	6	Survived
6	F	49	7 days	22.3	No	4.8	B2	II	PI	39	Survived
7	M	26	10 days	23.4	No	4.0	B2	II	Pneumothorax ^e^	4	Survived
8	F	45	8 months	21.4	No	17.0	B1	III	Pulmonary edema	10	Survived
9	M	39	1 month	24.7	Yes	5.1	B2	I	MC	7	Survived

^a^Masaoka stage; ^b^extra incubation days; ^c^mortality outcome during hospitalization; ^d^survived a secondary surgery; ^e^bilateral pneumothorax.

MC, myasthenic crisis; MODS, multiple organ dysfunction syndrome; PI, pulmonary infection.

**Table 3:** Association between clinical parameters and major adverse complications in logistic regression model

Parameters	95% CI^b^		*P*-value
	lower	upper	
Age (< 48 Vs. ≧ 48 years)	0.203	5.841	0.921
Gender (Male Vs. Female)	0.098	5.989	0.799
Smoking History (Yes Vs. No)	0.111	9.302	0.987
Course (< 234 Vs. ≧ 234 days)	0.130	6.497	0.933
BMI (< 23.0 Vs. ≧ 23.0)	1.179	44.564	0.033^*^
Tumor size (< 4.6 Vs. ≧ 4.6 cm)	0.531	14.790	0.225
WHO histology (Hyperplasia + A1 + AB Vs. B1 + B2 + B3)^a^	0.106	3.732	0.611
Masaoka stage (I + II Vs. III + IV stage)	0.158	9.968	0.829

^a^WHO histology, thymus hyperplasia, type A1, AB, B1, B2, B3 thymoma.

^b^confidence interval.

^*^P < 0.05.

**Figure 1:**
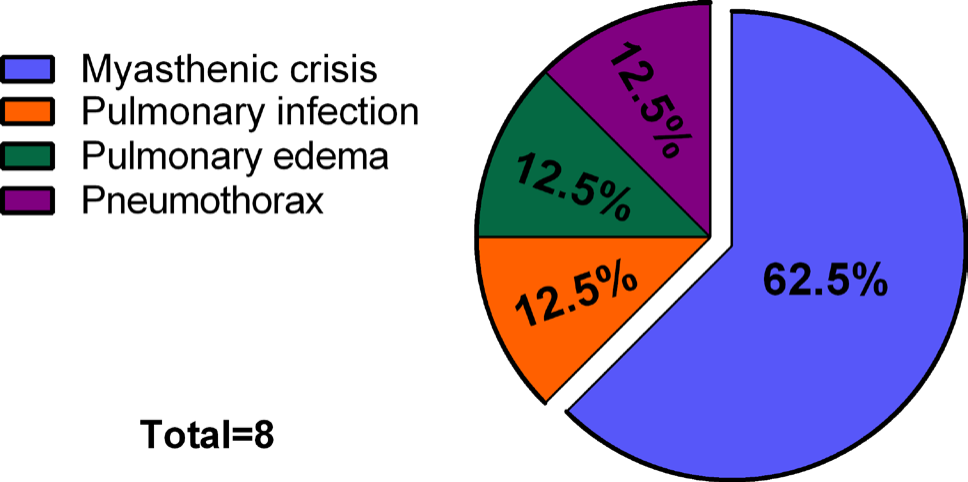
Causes of PRF

**Figure 2:**
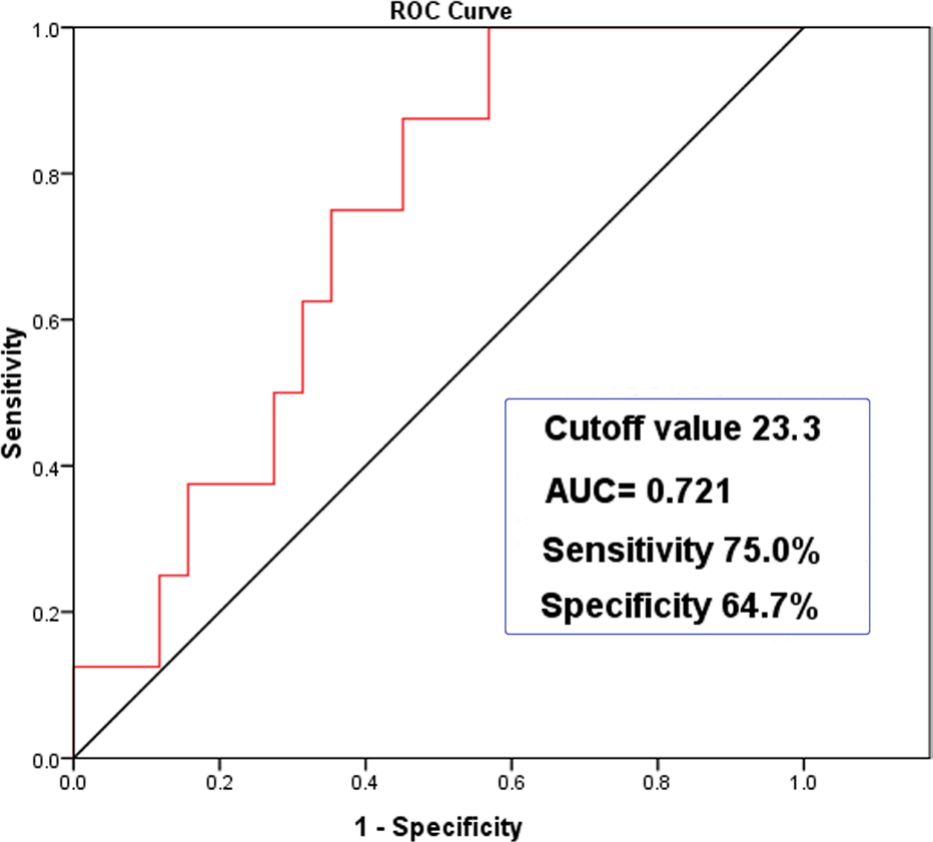
ROC curve analysis of BMI with PRF (95% CI 0.570–0.871, *P* = 0.046)

Considering the high incidence of PRF (13.6%) and subsequent threat to life, we further analyzed the reasons for PRF (Figure 1). Five cases were caused by postoperative myasthenic crisis, and there was one case each of pulmonary infection, bilateral pneumothorax, and pulmonary edema. The patient with pulmonary edema had a long disease history and giant tumor (17.0 cm). The high-BMI group had a significantly higher incidence of PRF compared with the low-BMI group (Table 4). Based on the receiver operating characteristic (ROC) curve, the cut-off BMI for PRF was determined to be 23.3 kg/m^2^. The area under the ROC curve (AUC) was 0.721 with a sensitivity of 75.0% and a specificity of 64.7% (95% CI 0.570–0.871, *P* < 0.05; Figure 2).

**Table 4:** The correlation between PRF and BMI

PRF	BMI (kg/m^2^)		χ^2^	*P*-value
	low group (< 23.0)	High group (≧ 23.0)		
Yes	2	6	4.035	0.045^*^
No	32	19		

^*^P < 0.05.

In addition to the major complications, other clinical monitoring parameters were evaluated (Table 5). These included: operative time (mean, 128 min); drainage time (mean, 4 d); total drainage volume (mean, 557 mL); and postoperative hospital stay (mean, 14 d). The postoperative hospital stay of patients in the high-BMI group was significantly longer than that of the low-BMI group (*P* = 0.005). Otherwise, the clinical monitoring parameters of the 2 groups were similar.

**Table 5:** Clinical monitoring parameters stratified by BMI

Monitoring parameters	BMI (kg/m^2^)		χ^2^	*P*-value
	low group (< 23.0)	High group (≧ 23.0)		
**Operation time (min)**			1.647	0.199
< 128	22	12		
≧ 128	12	13		
**Drainage days**			0.152	0.697
≧ 4	26	18		
> 4	8	7		
**Drainage volume (ml)**			0.136	0.712
< 557	22	15		
≧ 557	12	10		
**Postoperative hospital time (days)**			7.896	0.005*
< 14	30	14		
≧ 14	4	11		

^*^P < 0.05.

## DISCUSSION

In the current study, BMI was positively associated with major postoperative complications in patients with MG after transsternal thymectomy. Of particular note, these complications included PRF, which can be fatal, and which is caused by factors such as thymoma, drugs, pneumonia, surgery, and postoperative myasthenic crisis [19–21]. In the present study, the incidence rate of PRF was significantly higher in the patients of the high-BMI group compared with the low-BMI group (*P* = 0.045). Postoperative myasthenic crisis has a predominant role in PRF, with an incidence of 6–34% [21–23]. Similarly, myasthenic crisis occurred in 8.5% (5/59) of our patients. Thus, special monitoring care must be planned for overweight or obese MG patients scheduled for thymectomy.

Other reasons also contribute to PRF after surgery in MG patients with high BMI. Hypoxia is a main driver of adipose tissue dysfunction in obesity [24, 25], and oxygen tension activates hypoxia inducible factor-1 (HIF-1), a key factor in the response to low oxygenation, which in turn generates a series of inflammatory responses [6, 26]. There is no doubt that both hypoxia and systemic inflammation have adverse effects on MG. In addition, obesity is generally considered a high risk factor for postoperative complications [16–18]. This may partially be due to a raised diaphragm, increased chest pressure, and impaired pulmonary function. Hypoxia of the body can also be caused by MG itself, where respiratory muscles are affected. Accordingly, it is logical that a high BMI is tightly linked with PRF, as found in the present study. Furthermore, according to the ROC curve, the optimal cutoff value of BMI for PRF was determined to be 23.3 kg/m^2^, with a sensitivity of 75.0% and a specificity of 64.7%.

Besides major adverse complications, the present study showed that the postoperative hospital stay of patients in the high-BMI group was significantly longer than that of the low-BMI group. That may be a result of higher complication rates in high-BMI group. However, the groups were comparable with regard to operative time, days of drainage, and total drainage volume.

Excessive weight has attracted global attention, not only for its increased incidence, but for its association with cardiovascular disease, diabetes, and many cancers [2–4]. Positive dose-response links between weight and cancer risk have been demonstrated, with ∼1.2 to 1.5-fold and 1.5 to 1.8-fold higher risks for overweight and obesity, respectively [1]. High BMI indicates a poor prognosis in breast, colon, liver, and prostate cancer [8, 27–31]. Besides serving for lipid storage, adipose tissue is now recognized as an important endocrine organ [32, 33], secreting hormones such as adiponectin, leptin, interleukin (IL)-6, tumor necrosis factor α, IL-1β, and estrogen, most of which are linked to tumor proliferation, angiogenesis, invasion and metastasis [6–8]. Recent research suggests that DNA methylation changes as BMI increases [34].

There are several limitations in the present study. First, the data was retrospectively collected in a single center, and the sample size was limited. Given that, more samples and further investigation are necessary to expand on these findings in the future. Secondly, the BMI standard that is specific for Asian populations was adopted in this research, which has a lower threshold for overweight and obesity compared to the WHO classification [1, 35]. It remains unclear whether this biased the results, since theoretically, the higher the BMI, the more severe the dysfunction and chronic inflammation induced by adipose tissue. Finally, although BMI is considered a measure of adiposity, not all overweight or obese people demonstrate impairment, and some individuals with normal weight are impaired [36]. Therefore, further biomarkers are needed in addition to clinical measures.

## MATERIALS AND METHODS

### Ethics

The Ethics Committee of First Affiliated Hospital of China Medical University approved this retrospective study. The research was performed in accordance with the guidelines of the Ethics Committee, and conformed with the Declaration of Helsinki.

### Patients

We conducted a retrospective analysis of the clinical data of 59 MG patients, who had undergone transsternal thymectomy at our institute from June 2011 to May 2016. Patients who had received video-assisted thoracoscopic surgery were excluded from this study, to prevent bias with regard to inflammatory response, hospital stay, and surgery-related complications [37].

BMI was defined as the patient’s weight divided by height squared (kg/m^2^). According to the standard criteria for Asian-Pacific populations, normal, overweight, and obese was defined as BMI < 23.0, 23.0–24.9, and ≥ 25.0 kg/m^2^, respectively [38, 39]. Accordingly, the study population was stratified as either low or high BMI (< 23.0 or ≥ 23.0 kg/m^2^).

MG was diagnosed based on clinical manifestations, edrophonium test, electromyography, and serum auto-antibody test [40]. Histological classification was in accordance with the revised World Health Organization (WHO), and clinical staging was based on the Masaoka system [10, 41].

### Statistical analysis

All statistical data was analyzed using SPSS (Chicago, IL, USA) software, version 17.0. Categorical variables were assessed using Pearson’s chi-squared (χ^2^) test, Fisher’s exact test, and analysis of variance (ANOVA). A ROC curve analysis was utilized to identify the optimal cutoff value of BMI for predicting PRF. A logistic regression model was applied to characterize associations between clinical parameters and major adverse complications. Statistical significance was established at *P* < 0.05.

## CONCLUSIONS

In conclusion, this study showed that a BMI in the overweight or obese range is positively associated with longer postoperative hospitalization and major adverse postoperative complications, especially PRF. The data supports that being overweight or obese is a risk factor for postoperative adverse situations in MG patients. Hence, appropriate care must be taken when transsternal thymectomy is necessary.

## Abbreviations

ANOVA: analysis of variance
BMI: body mass index
MG: Myasthenia gravis
PRF: postoperative respiratory failure
ROC: receiver operating characteristic
